# Notice of the Galium Tinctorum as a Remedy in Chronic Diarrhea

**Published:** 1849-03

**Authors:** Thomas C. Osborne

**Affiliations:** Erie, Alabama


Art. VI.—*Notice of the Galium Tinctorum as a remedy in Chronic Diarrhea.* In a letter to the Editor, by Dr. Thomas C. Osborne, of Erie, Alabama.

Enclosed you will find a specimen of a plant which, in this vicinity and in the neighborhood of Mobile, is creating a considerable sensation among the people on account of its reputed virtues in the cure of chronic diarrhea. The discovery of its curative properties is said to be due to an aged negro near Mobile, who was in the habit of prescribing it to persons afflicted with the disease in question for several years before it began to attain any notoriety; and its reputation now, I believe, is chiefly due to the successful administration of it in the case of General Desha, one of our Tennessee acquaintances, who, for a long time, wasted under the influence of chronic diarrhea, and who, despairing of a cure by medicine, had gone to Pascagola to try the effects of change of air.

Of course, all this is information for which I cannot vouch, not having seen the General, or any one from his section of country who had ever tried the remedy; but the story is in many mouths and doubtless has some foundation.

There is, however, an interesting case, that of a little girl, a near neighbor of mine, which goes to prove that the plant is possessed of astringent properties. The child had labored under chronic diarrhea since May last, and was not much benefitted by the remedies employed. Finally the galium tinctorum was prescribed; it has not been long since she commenced its use, but her parents already regard the cure complete, and declare that "she fattens a pound every day."

The plant is called here *diarrhea weed*, and is used in the form of a decoction.

Great alarm is excited here by the rapid approach of cholera, of which a good many cases are said to have occurred among the humble part of the population of Mobile, but not of a very malignant character.

Influenza is prevailing in almost every part of South Alabama, some cases of which, in my practice, from neglect have run into pleuritis and pneumonia of quite a violent character. The lancet and opium are my sheet-anchor in its treatment, all cases yielding readily to their use.

January 12th, 1849.

